# Major heme proteins hemoglobin and myoglobin with respect to their roles in oxidative stress – a brief review

**DOI:** 10.3389/fchem.2025.1543455

**Published:** 2025-02-25

**Authors:** Rajarshi Sil, Abhay Sankar Chakraborti

**Affiliations:** ^1^ Allied Scientific Products, Kolkata, India; ^2^ Department of Biophysics, Molecular Biology and Bioinformatics, University College of Science, University of Calcutta, Kolkata, India

**Keywords:** hemoglobin, myoglobin, oxidative stress, free iron, fenton reaction

## Abstract

Oxidative stress is considered as the root-cause of different pathological conditions. Transition metals, because of their redox-active states, are capable of free radical generation contributing oxidative stress. Hemoglobin and myoglobin are two major heme proteins, involved in oxygen transport and oxygen storage, respectively. Heme prosthetic group of heme proteins is a good reservoir of iron, the most abundant transition metal in human body. Although iron is tightly bound in the heme pocket of these proteins, it is liberated under specific circumstances yielding free ferrous iron. This active iron can react with H_2_O_2_, a secondary metabolite, forming hydroxyl radical via Fenton reaction. Hydroxyl radical is the most harmful free radical among all the reactive oxygen species. It causes oxidative stress by damaging lipid membranes, proteins and nucleic acids, activating inflammatory pathways and altering membrane channels, resulting disease conditions. In this review, we have discussed how heme-irons of hemoglobin and myoglobin can promote oxidative stress under different pathophysiological conditions including metabolic syndrome, diabetes, cardiovascular, neurodegenerative and renal diseases. Understanding the association of heme proteins to oxidative stress may be important for knowing the complications as well as therapeutic management of different pathological conditions.

## 1 Introduction

Heme proteins, a large class of metallo proteins with heme prosthetic group, play crucial roles in human physiology (Tsiftsoglou et al., 2006), including oxygen transport (hemoglobin), oxygen (O_2_) storage (myoglobin), antioxidation (peroxidases, catalases), electron transfer (cytochromes), signal transduction (guanylate cyclase), and metabolic processes as enzymes (cyclooxygenase, nitric oxide synthase, etc.) (Table 1).

**TABLE 1:** Function of major heme-proteins.

Function	Relevant heme-protein
Oxygen transport	Hemoglobin
Oxygen storage	Myoglobin, neuroglobin
Mitochondrial respiration	Cytochromes
Cellular metabolism	Cytochrome P450 enzymes
Antioxidant function	Glutathione peroxidase, catalase
Cellular signaling	Soluble guanylate cyclase
Immune regulation	Indoleamine 2,3-dioxygenase
Antimicrobial defense	iNOS, NADPH oxidase, myeloperoxidase
Vasodilation	eNOS, cyclooxygenase
Endothelial and vascular integrity	eNOS

**FIGURE 1:**
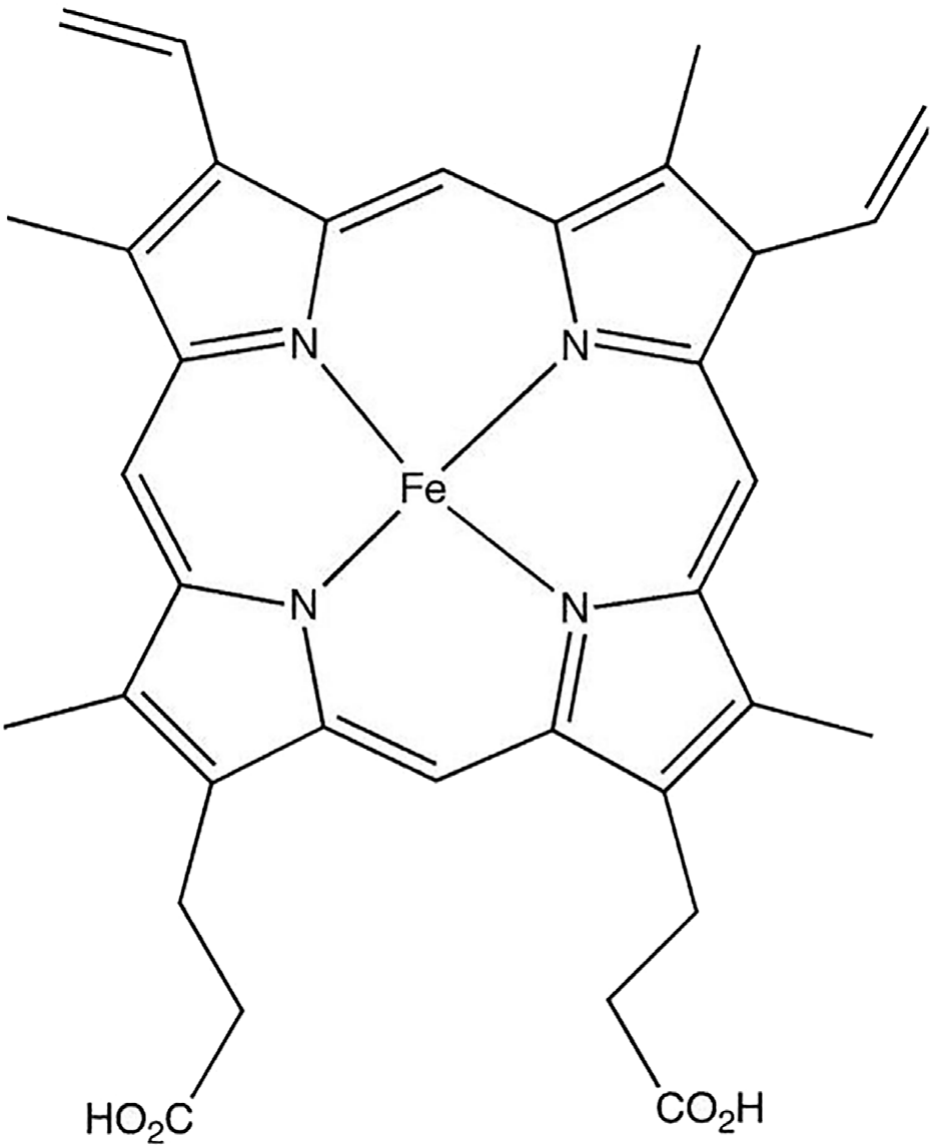
Structure of heme group.

Heme group consists of a central iron cation bound within a planar ring called protoporphyrin IX (Figure 1). The ring is made up of four pyrrole groups that are joined together by methine bridges. The iron is coordinated by four nitrogen atoms from the protoporphyrin ring. Among the two axial positions of iron, one position is available to bind with amino acid residue from the protein, usually a histidine. The other axial position remains free to bind with molecules like O_2_. This structure gives heme proteins their abilities to bind oxygen and participate in redox reactions (Smith et al., 2010; Ahmed et al., 2020). The iron in heme moiety typically switches between ferrous (Fe^2+^) or ferric (Fe^3+^) state (Kumar and Bandyopadhyay, 2005). Although Fe^2+^ and Fe^3+^ states are most common within the heme structure, oxidative states of heme-iron can vary from Fe^2+^ to Fe^5+^ (Karpefors et al., 2000; Dey and Ghosh, 2002).

Most of the oxygen, consumed during cellular respiration, is finally reduced to water molecule (H_2_O) through a four-electron transfer reaction catalyzed by cytochrome oxidase in complex IV of mitochondrial electron transport chain (ETC) (Wilson, 2017). The reaction is coupled with oxidative phosphorylation to produce adenosine triphosphate (ATP). However, a small portion of O_2_ undergoes partial reduction not only in the respiratory chain, but also during other physiological activities, such as phagocytosis, immune activation and xenobiotics metabolism (Galaris and Pantopoulos, 2008). This leads to formation of potentially harmful intermediates, collectively known as reactive oxygen species (ROS) including superoxide anion (O_2_^.-^), hydrogen peroxide (H_2_O_2_), hydroxyl radicals (^.^OH) etc (Table 2). To protect cells from ROS-mediated oxidative damages, several substances called antioxidants are absolutely essential. Under physiological condition, a robust antioxidant mechanism, comprising both enzymatic and non-enzymatic pathways, maintains tight balance between ROS generation and elimination (Kozlov et al., 2024). Oxidative stress, generated due to relative excess of ROS when compared with cellular antioxidants, has been linked to different disease conditions, namely, metabolic syndrome (Martemucci et al., 2023; Masenga et al., 2023), diabetes mellitus (Wronka et al., 2022), cardiovascular disease (Jin and Kang, 2024), neurodegenerative disease (Olufunmilayo et al., 2023), renal dysfunction (Ho and Shirakawa, 2023) and many other pathologies.

**TABLE 2:** Different reactive oxygen species (ROS).

ROS	Production
Superoxide anion radical (O_2_^.−^)	Generated in mitochondrial ETC
Hydrogen peroxide (H_2_O_2_)	By-product of different metabolic pathways
Hydroxyl radicals (^.^OH)	Generated by Fenton reaction between H_2_O_2_ and Fe^2+^ ion
Hydroxyl ion (OH^−^)	Generated by Fenton reaction between H_2_O_2_ and Fe^2+^ ion
Peroxide ion (O_2_^2−^)	Generated in mitochondrial ETC
Nitric oxide (^.^NO)	Synthesized endogenously by nitric oxide synthase (NOS) isoenzymes
Peroxynitrite (ONO_2_^−^)	Resulting from the reaction of superoxide and nitric oxide

Transition metals are able to catalyze the reduction of H_2_O_2_, a secondary metabolite, to highly reactive hydroxyl radical (^.^OH). This is especially prominent with metals like iron (Fe) and copper (Cu), which can readily change the oxidation states (Collin, 2019). Iron is considered as the most biologically relevant transition metal in this regard due to its high concentration in the human body (Song et al., 2022). Because of their diverse biological functions and widespread abundance, heme proteins are among the most studied biomolecules. However, being a major group of proteins in our system, heme proteins, due to their iron-containing porphyrin ring, are also responsible for ROS generation in different pathological conditions (Drvenica et al., 2022; Wilson and Reeder, 2022). In this review, we have discussed pathophysiology of two major heme proteins, hemoglobin and myoglobin, based on their roles in oxidative stress. Transition of hemoglobin between oxy and deoxy form (both in ferrous state) facilitates the transportation of oxygen in different tissues, while methemoglobin or ferrihemoglobin is not capable of oxygen transport. On the other hand, myoglobin, with its higher affinity for oxygen, is involved in oxygen storage in cardiac and skeletal muscle tissues. Besides their important biological functions to maintain cell health, hemoglobin and myoglobin can also contribute to oxidative stress, understanding of which is important to know the disease pathology.

## 2 Free heme, a major source of redox-active iron

**FIGURE 2:**
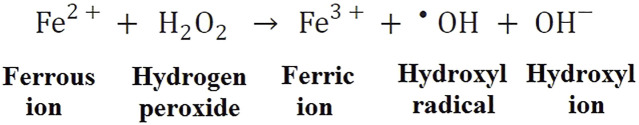
Fenton reaction. Ferrous (Fe^2+^) ion reacts with hydrogen peroxide (H_2_O_2_) to generate Ferric (Fe^3+^) ion, hydroxyl radical (^.^OH) and hydroxyl ion (OH^−^).

Heme prosthetic group of heme proteins is a major reservoir of iron in human body (Gozzelino et al., 2010). Iron homeostasis is tightly regulated to avoid accumulation of excess free heme (heme group not bound to any protein) (Sawicki et al., 2015). However, the free heme pool can increase under different pathological conditions like sickle cell anemia (Gbotosho et al., 2020), thalassemia (Ali et al., 2021), malaria (Ramos et al., 2024) and paroxysomal nocturnal hemoglobinuria (Gembillo et al., 2020). The underlying causes may be upregulation of heme synthesis, excess hemolysis or myolysis, elevated heme protein degradation, compromised integration of heme into heme proteins, or impaired heme oxygenase activity (Voltarelli et al., 2023). Although all transition metals have the ability to reduce H_2_O_2_ to hydroxyl radical (^.^OH), iron is considered as the most biologically active in this regard, because of its high abundance in the human body (Kontoghiorghe et al., 2015). Free heme acts as a good source of ferrous (Fe^2+^) ion to generate hydroxyl radical (^.^OH) through Fenton reaction (Jomova et al., 2023). In this reaction, iron, in its lower oxidation state (Fe^2+^), reacts with H_2_O_2_ to produce hydroxyl radical (^.^OH), a highly reactive free radical, and itself is oxidized to a higher oxidation state (Fe^3+^) (Figure 2) (Sadrzadeh et al., 1984; Thomas et al., 2009).

Hydroxyl radical (^.^OH), due to its strong oxidizing property, is capable of severe oxidative damages of biomolecules (Collin, 2019). Heme-driven production of ROS is involved in the pathophysiology of several disorders by damaging lipid membranes (Su et al., 2019), proteins (Pilo et al., 2022) and nucleic acids (Carter et al., 2022), activating inflammatory pathways (Wei et al., 2024), and perturbing membrane channels (Miranda et al., 2023), among other toxic effects.

Free heme is hydrophobic in nature (Tolosano et al., 2010). Because of high degree of lipophilicity, it easily intercalates into phospholipid bilayer of cell membrane and organelles (Higdon et al., 2012). Within oxidizing environment of membrane, H_2_O_2_ from various sources (e.g., activated leukocytes) cleaves the heme ring and interacts with the free redox-active iron, leading to enhanced production of hydroxyl radical (Chiabrando et al., 2014). This promotes membrane damage by lipid peroxidation, resulting increased membrane permeability and ultimately leading to cell death (Ryter, 2021).

Further, acting as a potent hemolytic agent, free heme affects stability of red blood cell membrane due to ROS generation and oxidative damage (Deuel et al., 2016), causing release of hemoglobin. Cell-free hemoglobin from high-intensity hemolysis is primarily eliminated from blood by renal clearance (Bolisetty et al., 2017). Post-filtration, the progressive acidification of the urine accelerates hemoglobin oxidation, globin structural destabilization, and heme release (Balla and Zarjou, 2021). Thus, heme-mediated oxidative stress and intravascular hemolysis of red blood cells are related to acute kidney injury (Vallelian et al., 2022).

## 3 Heme protein-mediated oxidative stress under hyperglycemic condition

**FIGURE 3:**
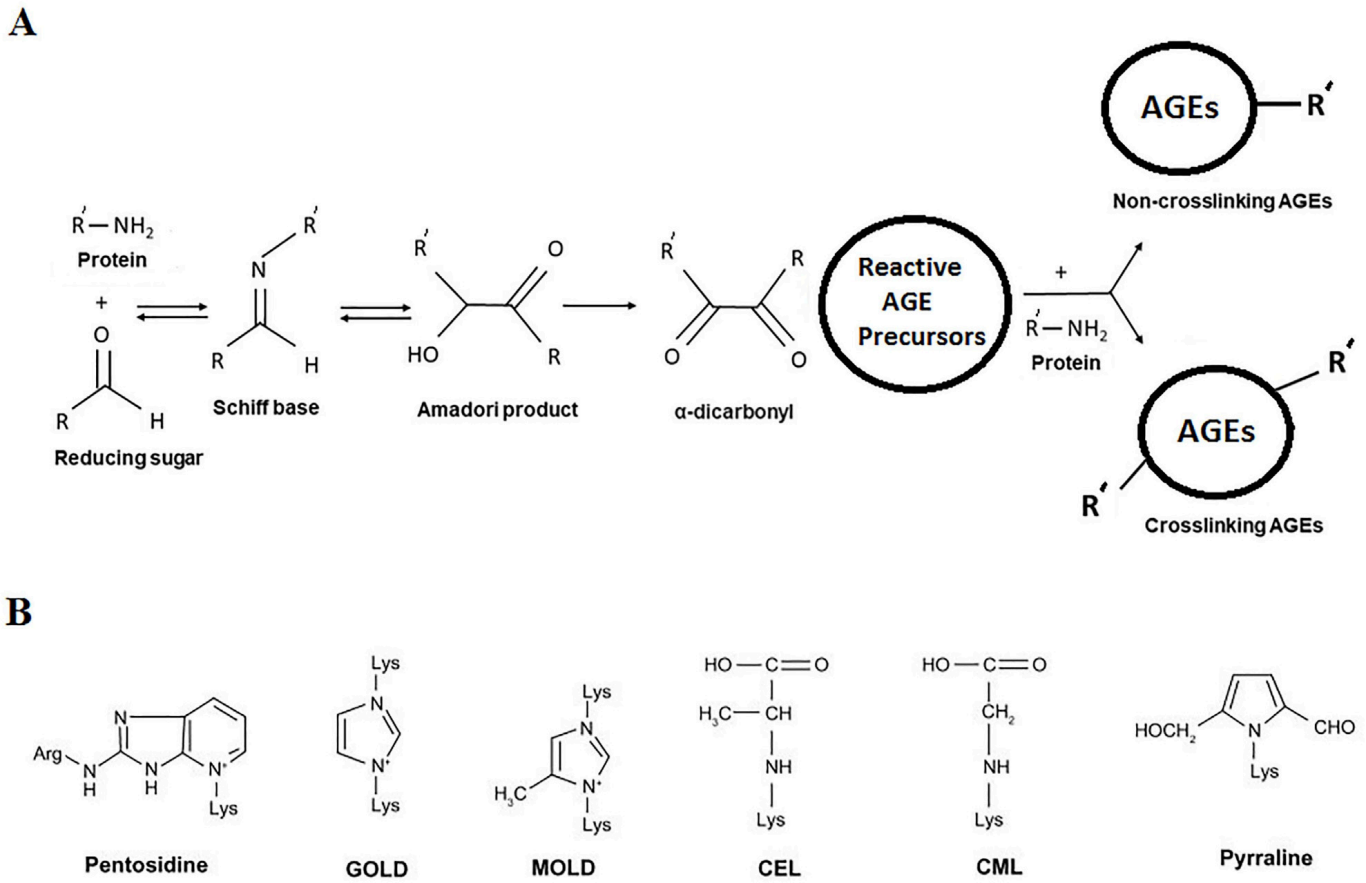
**(A)** Overview of non-enzymatic glycation (Maillard reaction) leading to formation of AGEs. **(B)** Structures of common AGEs.

Hyperglycemia is a condition characterized by elevated circulating blood glucose level. Persistent hyperglycemic state is the primary feature of metabolic syndrome (Cole and Florez, 2020) and diabetes (Minniakhmetov et al., 2024). Glucose and its oxidation by-products slowly but irreversibly react with the amino groups of long-life proteins (Khalid et al., 2022). The reaction is known as non-enzymatic glycosylation or glycation (also called Maillard reaction), leading to the formation of a heterogeneous set of compounds, known as advanced glycation end-products (AGEs) (Uceda et al., 2024). The sequence of non-enzymatic reactions leading to formation of AGEs has been shown schematically (Figure 3A). The reaction occurs when a carbonyl group of a reducing sugar (glucose, fructose, etc.) is exposed to an amino group of protein, leading to Schiff base formation, followed by Amadori rearrangement and formation of Amadori product. The Amadori product undergoes irreversible oxidation, dehydration, enolisation, cyclisation, and fragmentation leading to the formation of reactive intermediate AGE precursors. Reactive AGE precursors interact with lysine or arginine residues of proteins to form AGEs. Several important AGEs are pentosidine, N (6)-carboxymethyl lysine (CML), N (6)-carboxyethyl lysine (CEL), glyoxal-lysine dimer (GOLD), methylglyoxal-lysine dimer (MOLD), pyrraline, etc. Their structures are shown in Figure 3B. A specific cell surface receptor for AGEs (RAGE) has been shown to mediate inflammatory signal transduction via activation of NFκB, and p21 Ras (Deepu et al., 2024; Rojas et al., 2024).

**FIGURE 4:**
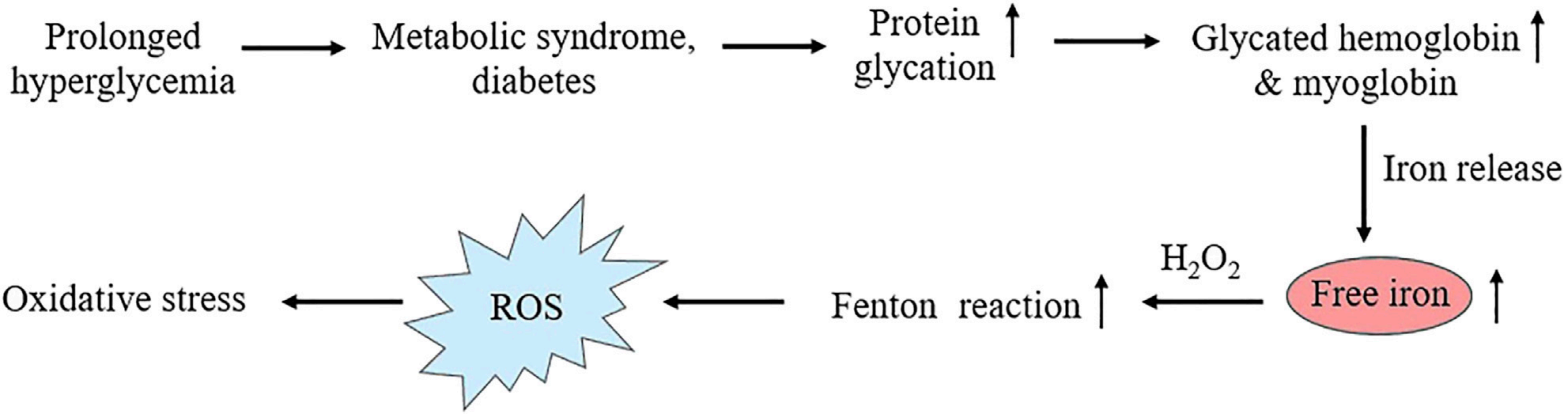
Schematic representation showing heme-protein glycation under hyperglycemic conditions, resulting enhanced glycated hemoglobin and myoglobin level followed by free iron release and Fenton reaction leading to oxidative stress.

Chronic hyperglycemia increases non-enzymatic glycation of proteins in metabolic syndrome and diabetes (Rabbani and Thornalley, 2021). Concentration of major glycosylated hemoglobin HbA_1c_, in which glucose is linked to N-terminal valine residues of β-chains increases proportionately with progression of hyperglycemia (Woleffenbuttel et al., 1996) and is used to monitor the extent or control of the disease condition. Several *in vitro* studies (Roy et al., 2004; Sen et al., 2005; Sen et al., 2007; Roy et al., 2010) have reported that, free iron release increases from the glycated form of major heme proteins, hemoglobin and myoglobin, resulting iron-mediated ROS generation by Fenton reaction. These findings are well supported by experimental studies in diabetic animals (Roy et al., 2008; Sen et al., 2011). Clinical studies on diabetic patients have reported a positive correlation between the serum free iron, glycated hemoglobin and fasting blood glucose (Kar and Chakraborti, 1999; Thomas et al., 2004; Zafar et al., 2011; Senghor et al., 2012; Achuthan and Mageswari, 2017). The sequence of hyperglycemia-associated protein glycation and free radical reactions has been shown in Figure 4.

In addition to glucose, several other reducing monosaccharides may initiate Maillard type reaction. For example, fructose is almost eight times more reactive than glucose (Bunn and Higgins, 1981). Like glucose, fructose (fructation) also induces structural and functional modification of hemoglobin leading to release of iron and iron-mediated oxidative reactions (Bose and Chakraborti, 2008). High concentration of fructose induces metabolic syndrome, which is characterized by insulin resistance, hyperglycemia, dyslipidemia and obesity (Taskinen et al., 2019). Animal model of this metabolic disorder also exhibits enhanced hemoglobin glycation, leading to increased iron release and oxidative reactions (Sil et al., 2013; Sil et al., 2015). Metabolic syndrome and diabetes are associated with elevated oxidative damage and inflammation (Darenskaya et al., 2021; Masenga et al., 2023). Free iron released from glycated hemoglobin leads to oxidative stress in these pathological conditions (Shetty et al., 2008).

In addition, a variety of highly reactive α-oxoaldehydes such as, 3-deoxy-glucosone, glyoxal and methylglyoxal are formed by auto-oxidation of glucose, Schiff base or Amadori products. The concentration of α-oxoaldehydes increases significantly in diabetic patients (Lapolla et al., 2003). These carbonyl intermediates can react again with free amino groups of proteins to form different AGEs (Thornally et al., 1999). Methylglyoxal has been reported to cause structural and functional modifications of heme proteins hemoglobin and myoglobin (Bose et al., 2013; Banerjee and Chakraborti, 2013; Banerjee et al., 2016).

If hyperglycemia is therapeutically controlled, heme protein glycation and free iron-mediated oxidative reactions are significantly reduced, as shown by using different phytoconstituents in experimental diabetes (Roy et al., 2008; Sen et al., 2011) and metabolic syndrome (Sil et al., 2013; 2015). Compared to chemical drugs, herbal therapeutic agents exhibit less or almost no side-effects during long-term uses (Jun et al., 2021; Kushwah et al., 2023; Guan et al., 2024). However, the limited solubility of most of the herbal components in aqueous media causing poor bioavailability restrict their therapeutic applications. To make them bioavailable, different techniques of nanonization have been developed (Hillaireau and Couvreur, 2009; Singh et al., 2017; Tran and Tran, 2019). Nanoformulations enhance the therapeutic potential of herbal agents in treatment of different diseases. Phytoconstituents in different nanocarriers exhibit better management of hyperglycemia and associated complications (Mukhopadhyay and Prajapati, 2015; Samaddar et al., 2017; Sun et al., 2020; Hou et al., 2021; Maity et al., 2022). Moreover, compared to free herbal agents, herbal agent-nanoparticle conjugates (entrapped, enveloped or tagged) appear to be more effective in preventing heme protein glycation and free iron-mediated oxidative reactions in experimental models (Bhattacherjee et al., 2016; Bhattacherjee et al., 2017; Roy et al., 2017; Mukhopadhyay et al., 2018; Maity and Chakraborti, 2020). These findings further confirm the role of heme proteins in hyperglycemia-associated oxidative stress.

## 4 Heme proteins and oxidative stress in cardiovascular diseases

Cardiovascular disease indicates problems in heart or blood vessels, including narrowing of the blood vessels in heart, other organs or throughout the body (Kim et al., 2023a). The condition is frequently associated in patients with chronic hyperglycemia (Rosengren and Dikaiou, 2023). Atherosclerosis is considered as the main underlying cause of cardiovascular disease (Poznyak et al., 2022). Oxidative stress, inflammation, endothelial dysfunction, and altered lipid metabolism are potential mechanisms leading to atherosclerosis (Jebari-Benslaiman et al., 2022). Oxidation of low-density lipoprotein (LDL) particles in the vascular endothelium has been reported to be an initial event in the atherosclerotic plaque formation (Khatana et al., 2020). The atherosclerotic plaques, especially the necrotic core of unstable plaques, contain apoptotic macrophages, erythrocytes and its metabolites - heme and hemoglobin (Li et al., 2006), and cytotoxic substances (cholesterol crystals, cholesterol esters, oxidized lipids, fibrin, inorganic minerals like hydroxyapatite, iron, and calcium) (Tong et al., 2023).

Several epidemiologic and experimental studies have shown an association between iron and atherosclerosis (Ma et al., 2022; Naito et al., 2022). Iron accumulates in the plaque either as inorganic or hemoglobin-bound iron (Vinchi et al., 2014; Vinchi et al., 2020). Heme-derived iron from hemoglobin can access the plaque upon intravascular hemolysis and intraplaque hemorrhage, affecting endothelial cells and macrophages (Turpin et al., 2021). In various types of cardiovascular diseases, impaired metabolism and exposure to heme occur in pathological processes, including neovascularization, internal hemorrhage, ischemia, and reperfusion (Guo et al., 2021). Free iron, heme and hemoglobin increase LDL oxidation, resulting enhanced sub-endothelial LDL retention favoring plaque progression (Vinchi et al., 2014; Michel and Martin-Ventura, 2020; Meegan et al., 2021).

The underlying mechanism is based on heme-iron accumulation, causing activation of multiple signaling pathways and impacting cell interactions within the atherosclerotic lesion (Choi et al., 2021; Gusev and Sarapultsev, 2023). Catalytically active iron is involved in producing ROS and promoting lipid peroxidation, which is crucial in the development of atherosclerosis. ROS generated by iron overload can damage DNA, proteins, and lipid structures in cell membranes, ultimately accelerating cardiomyocyte death (Drvenica et al., 2022). Thus, heme iron-mediated ROS generation plays an important role in physiological signaling pathways related to cardiovascular tissue injury and disease.

## 5 Heme protein-mediated oxidative stress in renal disease

Intravascular hemolysis, i.e., destruction of red blood cells is a fundamental feature of chronic hereditary and acquired hemolytic anemias (Kato et al., 2017), including those associated with hemoglobinopathies, complement disorders and vector-borne disease such as malaria (Schaer et al., 2013). Hemolysis results in the presence of excess amount of cell-free hemoglobin and heme in blood circulation, compared to the levels of their scavengers haptoglobin and hemopexin, respectively (Kumar and Bandyopadhyay, 2005). Free hemoglobin and heme present in plasma are filtered by the kidney, exposing the kidney to the injurious effects of heme and iron (Van-Avondt et al., 2019). Rhabdomyolysis can be induced by hereditary as well as acquired factors (Barbano et al., 2015; Stahl et al., 2020). The hereditary factors include metabolic myopathies occurring due to disorders of fatty acid oxidation, glycogen metabolism, purine nucleotide cycle, muscular dystrophies, calcium influx and caveolinopathy, etc. On the other hand, excess physical activity, influence of extreme temperatures, crush injury and trauma, vascular ischemia, drug toxicity, infections and sepsis, endocrine disorders, hyperthermia, electric current, toxins and alcohol, etc., may contribute as acquired factors leading to rhabdomyolysis. It is a clinical syndrome caused by skeletal muscle damage and release of its breakdown products including myoglobin into the circulation, followed by myoglobinuria and acute kidney injury (Gupta et al., 2021). Thus, both hemolysis and myolysis cause exposure of kidney to free heme proteins - hemoglobin and myoglobin (Van-Avondt et al., 2019; Nath et al., 2022).

High renal oxygen demand is associated with tubular oxygen consumption which is necessary for solute reabsorption (Bullen et al., 2017). In this oxygen-rich environment, H_2_O_2,_ present in urine, promotes oxidation of heme proteins resulting conversion of ferrous (Fe^2+^) iron to ferric (Fe^3+^) state, accompanied by the generation of superoxide radical. Further oxidation of heme proteins causes redox cycling between ferric (Fe^3+^) and ferryl (Fe^4+^) forms, finally leading to heme degradation and iron release (Nath et al., 2022). Free iron then increases hydroxyl radical generation by Fenton reaction (Andrianova et al., 2020; Rashid et al., 2023). Moreover, urinary acidification heightens lipid peroxidation caused by ferryl (Fe^4+^) -form of heme proteins and the accompanying generation of the potent renal vasoconstrictor, isoprostanes (Karamouzis et al., 2008). Isoprostanes are prostaglandin-like compounds that are generated by free radical-induced oxidation of membrane arachidonic acids (Milne et al., 2011). Excessive plasma and urinary isoprostanes are established biomarkers of oxidative stress in humans with chronic kidney disease (Granick et al., 2021). The findings suggest that heme protein-induced oxidative stress may act as a key mediator in acute renal injury as well as chronic kidney dysfunction.

## 6 Heme proteins and oxidative stress in neurodegeneration

Neurodegeneration is a complex process resulting in progressive and selective loss of neuronal functions (Katsnelson et al., 2016). Oxidative stress (Kim et al., 2015), protein aggregation (Sweeney et al., 2017), mitochondrial dysfunction (Bustamante-Barrientos et al., 2023) and endoplasmic reticulum stress (Ghemrawi and Khair, 2020) are well established pathways driving neurodegenerative processes.

Several findings suggest that dysfunction in iron and heme metabolism plays a crucial role in Parkinson’s disease and other neurodegenerative disorders (Carocci et al., 2018; Chiabrando et al., 2018; Ndayisaba et al., 2019). Neurodegeneration is often triggered by intracerebral hemorrhage (Schrag and Kirshner, 2020; Watson et al., 2022). The toxic properties of heme in the brain have been observed in intracerebral hemorrhage (Bulters et al., 2018; Pandya et al., 2021; Vasconcellos and Pimentel-Coelho, 2022). The intra-cerebral hemorrhage occurs with a bleeding event and the extravasation of blood components into brain parenchyma (Wu et al., 2002). With time, extravasated erythrocytes are lysed, releasing cytosolic components in the brain, including huge amounts of hemoglobin (Wagner et al., 2003). In the highly oxygen-rich environment of the brain, free hemoglobin in extracellular spaces undergoes oxidation and releases heme as well as iron in their free form. Iron has an inflammatory and pro-oxidative potential with the ability to activate the inflammasome, promoting oxidative stress, lipid peroxidation, inflammatory response and finally cell death (Righy et al., 2018; Sun et al., 2022). The involvement of iron in neurodegeneration has been well documented (Dusek et al., 2016; Ashraf et al., 2018), and iron chelation has been proposed as a therapeutic option in this disorder (Kupershmidt and Youdim, 2023; Marupudi and Xiong, 2024). It has been reported that intracellular iron-overload contributes to neuronal cell death via apoptosis and ferroptosis pathways (Zeng et al., 2021), while desferrioxamine (DFO), a well-known iron chelator, inhibits ferroptosis in Parkinson’s disease cell model improving expression levels of glutathione peroxidase 4 (GPX4) and ferritin heavy chain. Deferasirox (DFX), a trivalent iron chelator, exerts ameliorative effect in animal models of Alzheimer’s disease and tauopathy (Kwan et al., 2022). Another iron chelating drug, deferiprone, effectively improves patient’s condition in Parkinson’s disease (Martin-Bastida et al., 2017; Negida et al., 2024).

## 7 Heme-proteins and ferroptosis

Ferroptosis is a relatively new form of programmed cell death (Dixon et al., 2012). It is characterized by iron-dependent accumulation of ROS and peroxidation of polyunsaturated fatty acids of membrane phospholipids. It is different from other cell death modalities in many aspects. Cells that undergo ferroptosis have morphological, biochemical, genetic, and metabolic features distinct from those of previously identified programmed cell deaths, such as apoptosis, pyroptosis, entosis, mitoptosis, necroptosis, and autophagy (Lin et al., 2022). Ferroptosis is triggered by excessive peroxidative damage of membrane lipid bilayer due to labile iron overload causing Fenton reaction-mediated hydroxyl radical generation and lipid peroxidation, and compromised antioxidant defense systems, including reduced glutathione (GSH)/GPX4-dependent and independent pathways (Dixon and Pratt, 2023; Xu et al., 2024). Recent findings suggest that ferroptosis plays a key role in pathogenesis of diabetes (Sha et al., 2021; Liu et al., 2024a), cardiovascular disease (Wang et al., 2021a; Zhang et al., 2022), kidney disease (Martin-Sanchez et al., 2020; Wang et al., 2023), non-alcoholic fatty liver disease (Wang et al., 2022; Zhao et al., 2023), neurodegenerative disorders (Lane et al., 2021; Ryan et al., 2023) and tumor progression (Gong et al., 2022; Kim et al., 2023b). Release of free iron from heme proteins, especially hemoglobin (Cao and Dixon, 2016; Liu et al., 2024b) and myoglobin (Luan et al., 2023; Qiao et al., 2023) under different pathophysiological conditions may promote Fenton reaction and oxidative stress leading to ferroptosis. Iron chelators effectively prevent the occurrence of ferroptosis, which may be an effective approach for the treatment of iron-related disorders (Chen et al., 2020; Pei et al., 2022; Liu et al., 2024a). FerroTerminator1, a novel iron chelator, has been reported to ameliorate liver damage by inhibiting hepatic iron accumulation and ferroptosis in various metabolic dysfunction-associated steatohepatitis (MASH) (Tao et al., 2024).

## 8 Molecular basis of heme protein-mediated oxidative stress in pathological conditions

Iron is an essential component of heme proteins regulating several biochemical functions. However, free redox-active iron, can be harmful for cells by promoting oxidative stress. Iron metabolism is, therefore, tightly regulated to fulfill the demand for heme protein biosynthesis as well as by avoiding detrimental effect of the redox-active iron (Gozzelino and Arosio, 2016; Muckenthaler et al., 2017). Under normal physiological condition, iron balance is finely controlled via binding to proteins, namely, transferrin (involved in iron transport) and ferritin (responsible for iron storage) (Kawabata, 2022). However, in various pathological conditions, as discussed in earlier sections, generation of free redox-active iron exerts adverse effects.

In diabetes, iron release increases from glycated hemoglobin resulting ROS production. Increased generation of ROS induces lipid peroxidation. Cell membranes and organelle membranes are especially sensitive to ROS damage due to high content of polyunsaturated fatty acids. Lipid peroxidation and accumulation of peroxidation products are the main risk factors of diabetes-induced vascular dysfunction (Negre-Salvayre et al., 2010). The markers of lipid peroxidation include malonaldehyde (MDA), hydroxynonenal (HNE), and 8-isoprostaglandin F2⍺ (Gopaul et al., 1995; Gaweł et al., 2004). 8-isoprostaglandin F2⍺ exhibits multiple activities to induce vascular dysfunction, including plateles adhesion and aggregation as well as vasoconstriction. Growing evidence has suggested that oxysterols (lipid peroxidation products of cholesterol) are involved in the pathology of diabetes mellitus (Samadi et al., 2021). Oxysterols are also found elevated in the brains of diabetic rodent models and in the blood of diabetic patients (Weigel et al., 2019). Increased levels of oxysterols were also found in the plasma and vascular walls of patients with cardiovascular diseases, particularly in atherosclerotic lesions (Vejux and Lizard, 2009). Macrophages absorb excessive oxysterols in the presence of high level of peripheral cholesterol. Accumulation of these cholesterol-rich immune cells on blood vessel walls contributes to vascular dysfunction and atherosclerosis (Chistiakov et al., 2016). Under diabetic condition, excess ROS, thus, plays a critical role in the occurrence and development of cardiovascular diseases (Wang et al., 2021b).

Protein carbonylation is one of the most detrimental oxidative protein modifications, which are not easily reversed (Wong et al., 2013). It is also regarded as a crucial biomarker of oxidative stress-related diseases (Cattaruzza, and Hecker, 2008). ROS can oxidize amino acid side groups of protein to introduce carbonyl group at specific sites, which leads to loss of catalytic or structural function of the modified proteins (Hecker and Wagner, 2018). For example, carbonylation of actin leads to changes in cytoskeleton dynamics and damage of barrier function of blood vessels (Dalle-Donne et al., 2001). Moderately carbonylated proteins are susceptible to degradation by the proteasomal system. However, heavily carbonylated proteins tend to form aggregates that are resistant to proteolytic degradation, and accumulate as damaged or unfolded proteins (Dalle-Donne et al., 2006). A large number of neurodegenerative diseases are directly associated with the accumulation of proteolysis-resistant aggregates of carbonylated proteins in tissues (Gonos et al., 2018).

Besides induction of tissue oxidative stress damage, ROS can also trigger the aggregation of inflammatory cells, and formation of inflammatory cytokines related to various pathological processes. ROS-mediated activation of various inflammatory signaling pathways including nuclear factor kappa B (NF-κB) signaling, Janus kinase/signal transducers and activators of transcription (JAK/STAT) signaling, and mitogen-activated protein kinase (MAPK) signaling, have been reported in diabetes and associated complications (An et al., 2019; Chen et al., 2021; Lei et al., 2021). ROS can directly attack the free sulfhydryl (-SH) groups, which are necessary to maintain protein folding. It thus induces oxidative modification of proteins, and triggers endoplasmic reticulum (ER) stress due to the prolonged accumulation of unfolded or mis-folded proteins in the ER lumen (Lenna et al., 2014; Zeeshan et al., 2016). Studies have shown that elevated ROS and ER stress can lead to endothelial dysfunction in hyperglycemic condition (Kapadia et al., 2021; Chen et al., 2022).

## 9 Conclusion

**FIGURE 5:**
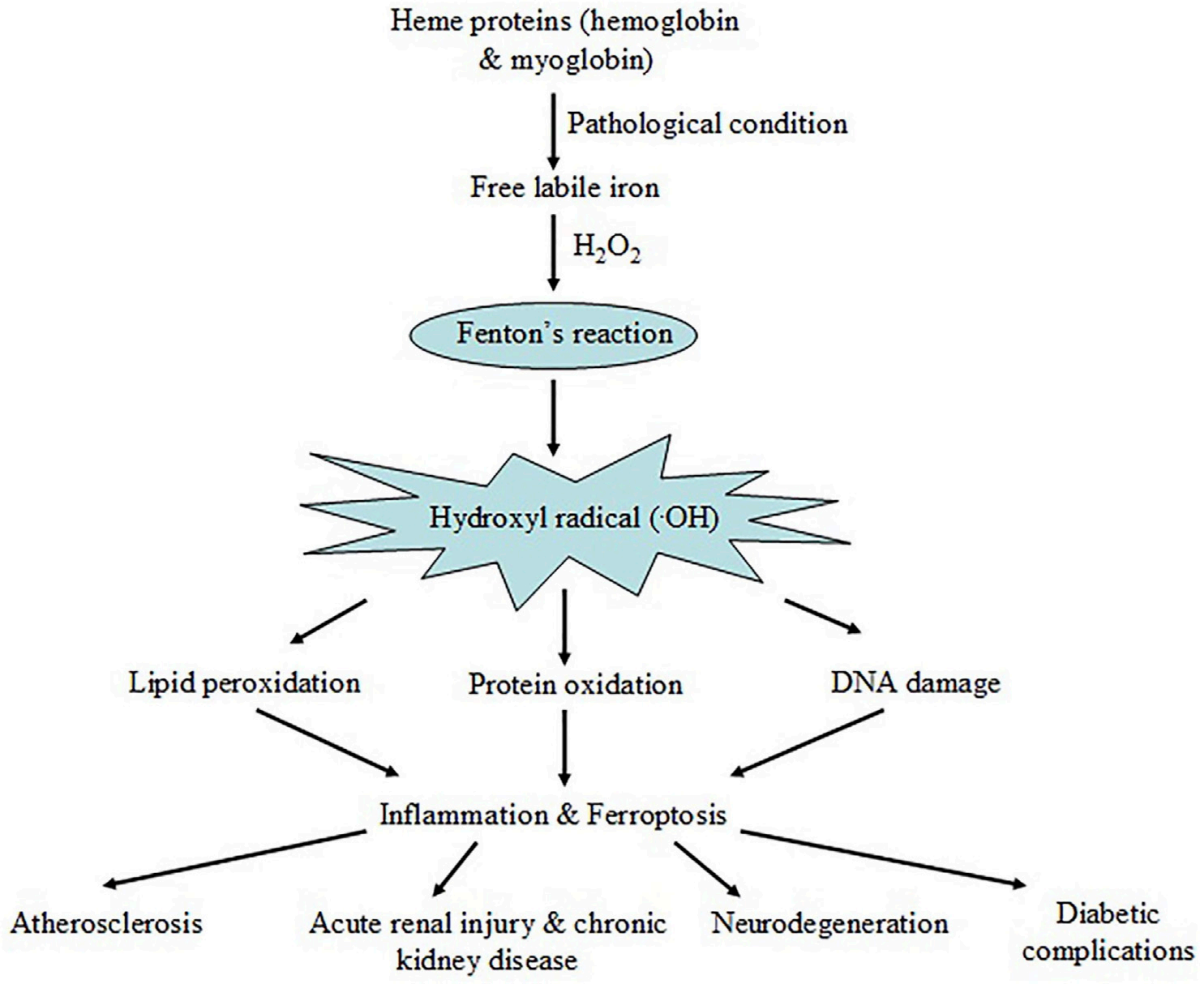
Schematic representation showing iron release from major heme-proteins, hemoglobin and myoglobin, under pathological conditions resulting Fenton reaction and ROS generation. The event leads to oxidative damage of cellular macromolecules triggering ferroptosis and inflammation causing disease complications.

We have discussed, in brief, the potential mechanism leading to hemoglobin and myoglobin-mediated oxidative stress in various disease conditions. Under pathophysiological stress, heme and free iron release from major heme proteins, hemoglobin and myoglobin, leads to harmful hydroxyl radical (^.^OH) generation via Fenton reaction. Hydroxyl radicals (^.^OH) cause oxidative damage of different cellular components by lipid peroxidation, protein carbonylation and DNA damage. Such events may be associated with ferroptosis and inflammation causing atherosclerosis, renal injury, neuronal cell damage and hyperglycemia-related complications (Figure 5).

Iron is not only an essential cofactor for vital biochemical activities in human physiology, but also a potential biohazard. Cells have evolved stringent mechanism to control iron metabolism and to satisfy metabolic needs, minimizing the risk of iron toxicity. However, in pathophysiological condition, iron can contribute oxidative stress worsening the situation. An increasing number of experimental studies has provided evidence regarding involvement of heme iron in several disease progression. Hence a comprehensive understanding of the mechanism linking heme proteins to oxidative stress is potentially beneficial for future therapeutic intervention.
